# Effects of an individualized nutritional intervention on kidney function, body composition, and quality of life in kidney transplant recipients: Study protocol for a randomized clinical trial

**DOI:** 10.1371/journal.pone.0272484

**Published:** 2022-08-04

**Authors:** Tássia Louise Sousa Augusto de Morais, Karla Simone Costa de Souza, Mabelle Alves Ferreira de Lima, Maurício Galvão Pereira, José Bruno de Almeida, Antônio Manuel Gouveia de Oliveira, Karine Cavalcanti Mauricio Sena-Evangelista, Adriana Augusto de Rezende

**Affiliations:** 1 Postgraduate Program in Nutrition, Center for Health Sciences, Federal University of Rio Grande do Norte, Natal, Rio Grande do Norte, Brazil; 2 Department of Clinical and Toxicological Analysis, Federal University of Rio Grande do Norte, Natal, Rio Grande do Norte, Brazil; 3 Division of Nephrology, Department of Integrated Medicine, Federal University of Rio Grande do Norte, Natal, Rio Grande do Norte, Brazil; 4 Postgraduate Program in Health Sciences, Center for Health Sciences, Federal University of Rio Grande do Norte, Natal, Rio Grande do Norte, Brazil; 5 Department of Nutrition, Federal University of Rio Grande do Norte, Natal, Rio Grande do Norte, Brazil; Harvard Medical School, UNITED STATES

## Abstract

**Background:**

Proteinuria after kidney transplantation (KTx) has been a frequent problem due to several factors, high protein intake being one of them. Individualized nutritional intervention in the late post-KTx period can promote the improvement or the reduction of risks associated with the parameters of evaluation of kidney function, body composition, and quality of life in individuals submitted to KTx.

**Methods:**

This is a single-center, randomized and stratified clinical trial. The study will be conducted in a university hospital in northeastern Brazil with 174 individuals aged ≥19 years submitted to KTx and followed up for 12 months. Assessments will take place at 3-month intervals (T0, T3, T6, T9, and T12). The patients will be allocated to intervention and control groups by random allocation. The intervention group will receive individualized nutritional interventions with normoproteic diets (1.0 g/kg) after 60 days of KTx whereas the controls will receive the standard nutritional guidance for the post-KTx period. The primary efficacy variable is the change from baseline in log proteinuria assessed with the urinary albumin/creatinine ratio. Secondary efficacy variables include body composition, anthropometry, quality of life assessment and physical activity, lipid profile and glycemic control. Ninety-two subjects per group will afford 70% power to detect a difference of 25% between groups in log proteinuria. Primary efficacy analysis will be on the modified intention-to-treat population with between-groups comparison of the change from baseline in log proteinuria by analysis of covariance.

**Discussion:**

The study will assess the effects of an individualized nutritional intervention on proteinuria 12 months after KTx.

**Trial registration:**

REBEC (RBR-8XBQK5).

## Introduction

Nutritional interventions in all stages of kidney transplantation (KTx) play important roles in individual care and in preventing common comorbidities that may arise in the post-KTx period, such as obesity or malnutrition, hypertension, diabetes, cardiovascular diseases [1], and clinical conditions such as proteinuria [2].

Proteinuria is an indicator of kidney function and, when present, it is associated with a higher incidence of acute rejection, hypertension, graft dysfunction, cardiovascular risk, and long-term mortality [3, 4]. This clinical condition may be due to several factors, such as the nephrotoxicity of immunosuppressive therapy, recurrent glomerulonephritis, glomerulopathies, ischemic damage from surgery, diabetic or hypertensive nephropathy, presence of native kidneys, and other indirect factors such as high sodium and protein intakes in the diet [2].

Although this perception exists, there are few studies that have assessed the impact of dietary protein in the late post-KTx period (> 6th week after surgery), making it difficult to construct guidelines for dietary recommendations for the nutritional management of these patients based on scientific evidence [5, 6]. Currently, only generalized nutritional guidelines are known that focus on preventing complications after KTx [7, 8]. Studies that consider nutritional interventions are still limited and inconclusive, mainly because they do not suggest evidence of long-term results [6, 9].

In the immediate post-KTx period (< 6th week after surgery), a high-protein (1.3 to 2.0 g/kg/day) and hypercaloric (30 to 35 kcal/kg/day) diet should be recommended to meet the needs caused by the resultant metabolic stress after surgery and the use of high doses of immunosuppressive therapy [10]. However, in the late post-KTx period, the characteristics of the diet are changed to normoproteic (1.0 g/kg/day) and normocaloric (25 to 30 kcal/kg/day) in order to meet the individual needs of each patient [11]. In addition, the choice of foods to be recommended is conditioned to the risks of non-surgical complications, requiring strict control over blood pressure, lipid and glycemic profile, weight gain, and the influence that these variables have on patients’ quality of life [6, 8, 10, 12].

The average weight gain after the first year of surgery is close to 5–10 kg or 5%–10% of the initial body weight [13, 14]. Likewise, the body composition analysis shows a higher percentage of adiposity in relation to lean mass, which is associated with obesity and other factors [15], and may suggest a negative influence on kidney function, especially in the success of the graft [20]. In addition to immunosuppressive therapy, increased appetite, change in lifestyle and reduced dietary restrictions as well as reversal of uremia and limited physical activity contribute to this scenario [13].

The patient’s quality of life and renal graft survival depend directly on post-KTx clinical care. Multiprofessional follow-ups help in the maintenance and early identification of abnormalities or signs of kidney function regression. Possibly, adequate intake of energy and protein in the diet could contribute to control lipid levels and to maintain the balance of micronutrients such as sodium, calcium, iron, potassium and phosphorus in the late post-KTx period that favors the maintenance of kidney function, especially in the reduction or prevention of proteinuria, decreasing the risk of complications, helping to maintain a favorable body composition, and improving the quality of life of patients undergoing KTx [1, 2, 6, 10].

Considering the scarcity of nutritional guidelines and the heterogeneity of experimental studies on dietary recommendations after KTx, this study aimed to evaluate the effect of an individualized nutritional intervention on the parameters of assessment of kidney function, body composition, and quality of life in individuals submitted to KTx followed for 12 months in a nephrology clinic at an university hospital in northeastern Brazil.

## Materials and methods

This is a single center, stratified and randomized clinical trial to evaluate the efficacy of individualized nutritional intervention considering normal protein diet with consumption of 1.0 g/kg versus standard nutritional guidelines in chronic kidney disease patients submitted to KTx in the previous two months. The trial site will be the Onofre Lopes University Hospital/Federal University of Rio Grande do Norte (UFRN), Rio Grande do Norte, Brazil.

The study was submitted to and approved by the Research Ethics Committee of Onofre Lopes University Hospital/UFRN with authorization number 3,127,266 and registered with the CAAE: 02445018.7.0000.5292. Eligible patients will be informed verbally and in written about the objectives, procedures and potential risks of the study. Those agreeing to participate will be asked to sign an informed consent form. The clinical trial was registered on the *Brazilian Clinical Trials Registry (REBEC)* online platform, obtaining the registration RBR-8XBQK5 and UTN (*Universal Trial Number*) U1111-1231-6000, linked to the World Health Organization (WHO).

### Trial design

**Fig 1:**
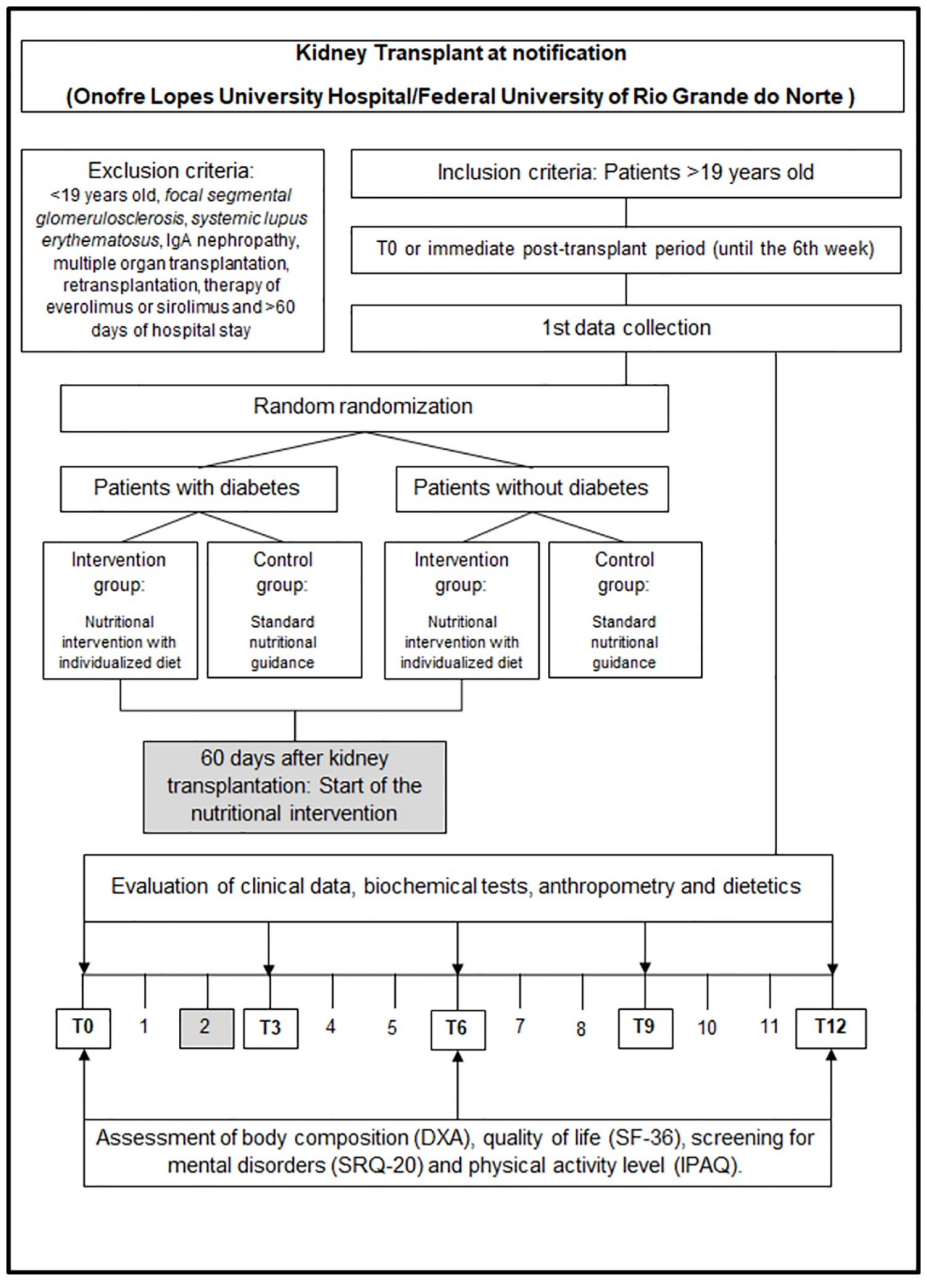
Flowchart in months with the design of the study from the recruitment of patients to data collection, considering the time of transplantation. DXA, *double-beam X-ray absorptiometry*; SF-36, *Medical Outcomes Study 36-Item Short-Form Health Survey*; SRQ-20, *Self-Reporting Questionnaire*; IPAQ, *International Physical Activity Questionnaire*.

**Fig 2:**
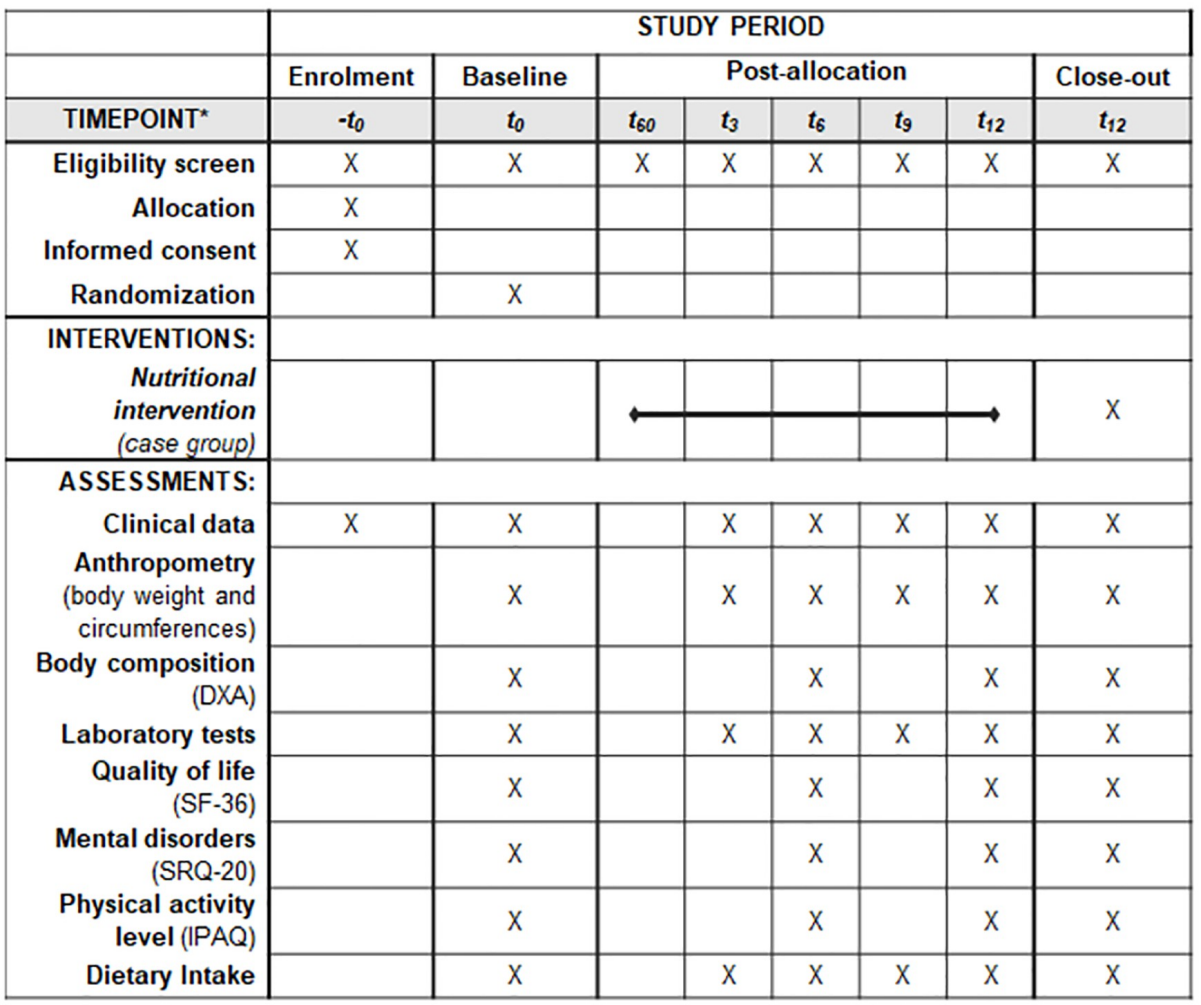
SPIRIT diagram. *Time-points of the protocol: -T0, enrolment; T0, baseline; T60, 60th day after transplant; T3, month 3; T6, month 6; T9, month 9; T12, month 12. DXA, *double-beam X-ray absorptiometry*; SF-36, *Medical Outcomes Study 36-Item Short-Form Health Survey*; SRQ-20, *Self-Reporting Questionnaire*; IPAQ, *International Physical Activity Questionnaire*.

The clinical trial groups will be evaluated at Time 0 (T0) or immediate post-KTX and every three months thereafter, until to complete 12-months post-KTx. At each study visit, the patients will be evaluated for kidney function and other biochemical parameters, food consumption, anthropometric measurements, body composition, and quality of life, as shown in Fig 1. Fig 2 depicts the study diagram according to the Standard Protocol Items: Recommendations for Interventional Trials–SPIRIT [16].

### Inclusion and exclusion criteria

The study will include individuals of both sexes aged 19 years and older who underwent KTx in the previous two months, with exclusively on oral feeding, and be literate or have a literate caregiver. The exclusion criteria will be patients with diagnosis of focal segmental glomerulosclerosis, systemic lupus erythematosus, IgA nephropathy, with multiple organ transplantation, with a history of recurrent transplantation due to chronic rejection, patients on immunosuppressive therapy of everolimus or sirolimus, and with hospital stay longer than 60 days after surgery.

### Primary and secondary efficacy variables

The primary and secondary efficacy variables defined for the study are described in Table 1.

**Table 1:** Description of the primary and secondary efficacy variables for patients who have received a KTx and participated in the study.

Primary efficacy variable	Secondary efficacy variable
**• Change from baseline in log proteinuria (urinary albumin/creatinine ratio)**	• Change from baseline in the body adiposity percentage
	• Change from baseline in lean body mass
	• Change from baseline in body mass index
	• Difference in quality of life and physical activity
	• Change from baseline in lipid profile (triglycerides, total cholesterol, high-density lipoprotein cholesterol and low-density lipoprotein cholesterol)
	• Change from baseline of glycemic control (fasting glucose and glycated hemoglobin).

### Sample size and recruitment

The sample calculation was performed using the software Stata 15.1 (Stata Corp., College Station, TX), based on a published study of patients who had undergone KTx with a mean log proteinuria of 2.36 mg (standard deviation of 1.55) at 6 months, by the urinary albumin/creatinine ratio [17]. To demonstrate a difference of 25% in the geometric mean of proteinuria at 6 months between the trial groups, with α error of 5% (α ≤ 0.05) and statistical power of 70%, 87 patients per study group will be needed. Assuming a drop-out rate of 5% prior to the first follow-up visit, the sample size is set at 92 subjects per group. Recruitment is scheduled to take place from May 1, 2019 through to December 31, 2022.

### Randomization

Randomization will be by permuted blocks of varying size and stratified by diagnosis of *Diabetes mellitus*. Two randomization lists, one for each stratum, will be generated from the *randomization*.*com* website [18]. From the randomization lists, two sets of sequentially numbered opaque envelopes will be created, containing the identification of the trial groups. As the nutritional intervention will be tailored to the treatment group, there is no possibility of blinding.

### Nutritional intervention

Participants randomized to the intervention group will be subjected to an individualized nutritional intervention prescribed after 60 days of KTx by a professional nutritionist participating in the research through an individualized plan with a two-option substitution rate calculated with the aid of the *Virtual Nutri Plus 2*.*0* software, using nutritional recommendations for the late post-KTx period, according to Table 2 [10, 11]. The foods and preparations on the plan will be presented in the form of weights and conventional homemade measures [19, 20] that the patients are familiar with in their routines. In order to gain a better understanding of the diet plan, photographic records of portions of food [21] and real-sized food models made of silicone will be used as illustrations for demonstrating the portions suggested in the menu.

**Table 2:** Nutritional recommendations for food planning in the late post-KTx period.

Nutrient	Nutritional recommendation
Energy	25–30 kcal/kg/day
Protein	1.0 g/kg
Total lipids	< 30% of the total kcal
Saturated fatty acids	< 10% of total calories
Monounsaturated fatty acids	10–15% of total calories
Polyunsaturated fatty acids	≥ 10% of total calories
Cholesterol	< 300mg
Carbohydrates	50% of total kcal
Fiber	25–30 g
Sodium	3–4 g
Potassium	Restricted (1–3g) only if hyperkalemia and/or oliguria are present
Phosphorus	1200–1500 mg
Calcium	800–1500 mg
Iron	The need for supplementation depends on the body reserves
Magnesium	Supplementation, if required, while using cyclosporine
Water-soluble vitamins	Usually no need for supplementation
Vitamin D3	1 a 2 μg, if required

Source: Martins; Pecoits-Filho; Riella [10]. Riella; Martins [11].

Patients randomized to the control group will receive nutritional guidance for the post-transplant period as offered by the hospital. The guidelines manual will be prepared based on literature [10, 11] and will have information and suggestions for weight and blood lipid and glucose control, attention to the intake of foods that are sources of fat, sodium and general guidelines on healthy, safe eating and food recipes.

### Treatment adherence

Patients who receive the nutritional intervention will be followed up in the same period as the patients in the control group, with adherence to the food plan being verified through a 3-day food record (applied during all evaluation periods) for the evaluation of food consumption. In addition, the frequency of individuals in face-to-face consultations will also be assessed during these meetings, and the difficulties and doubts of patients about the diet plan will be addressed.

Patients, who during the course of the clinical trial, have the introduction of mammalian target of rapamycin (mTOR) inhibitors (everolimus or sirolimus) in their immunosuppressive regimen, initiate a protein food supplementation, or who abandon and withdraw the consent to participate will have their trial prematurely terminated.

### Evaluation and analysis of biochemical parameters

The urinary albumin/creatinine ratio will be evaluated from the first morning urine samples to assess albuminuria. Urinary albumin and creatinine concentrations will be determined using Wiener kits using the CMD-800 (Wiener Laboratories, Rosario, Argentina).

Blood samples will be taken after a 12-hour fasting period. Blood biochemical parameters will be performed by means of urea, creatinine, uric acid, triglycerides, total cholesterol, high-density lipoprotein cholesterol, low-density lipoprotein cholesterol, as well as, fasting glucose, basal insulin, and glycated hemoglobin will be evaluated. Other parameters will be evaluated, such as serum concentrations of total proteins, albumin, total calcium, phosphorus, sodium, potassium, magnesium, total alkaline phosphatase activity, parathyroid hormone (PTH), 25-hydroxyvitamin D [25(OH)D] and tacrolimus concentrations.

The urea, creatinine, uric acid, triglycerides, total cholesterol, high-density lipoprotein cholesterol, low-density lipoprotein cholesterol, fasting glucose, glycated hemoglobin, total proteins, albumin, total calcium, phosphorus, sodium, potassium, magnesium, total alkaline phosphatase will be evaluated in blood samples using *Wiener* kits according to the methodology described by the manufacturer, using the *CMD-800* biochemical analyzer (*Wiener Laboratories*, *Rosario*, *Argentina*). Further, PTH, 25(OH)D, basal insulin and tacrolimus concentrations will be measured by the chemiluminescence method with ABBOTT^®^ kits, using the ARCHITECT i2000SR Immunoassay *Analyzer (ABBOTT Diagnostics*, *Illinois*, *USA*).

All biochemical analyzes will be performed at all the time points in the study (T0, T3, T6, T9, and T12 after KTx).

### Assessment of dietary intake

Dietary intake will be assessed using the 3-day food record method completed by the patient himself, if literate, or by a caregiver who follows their daily routine, for 3 consecutive days that include a weekend day. Information about the food consumed, the amount and details of the type of the preparation, the times and places of meals, and the consumption of vitamin, mineral, and food supplements, will be collected. In periods T0, T3, T6, T9, and T12 after KTx, all the patients will be instructed on how to fill in the requested information and, to facilitate collection, all of them will receive a manual of home measurements prepared by the authors with images of utensils and conventional measures of food. The 3-day food record annotation form was adapted to facilitate the filling of meal-related information, by including information in a “step by step” form for a better understanding by patients or companions (S1 Appendix).

The analysis of the food records will be made in a qualitative way by observing the type and quality of the food consumed. The quantity of each food item and drink will be converted into grams or milliliters using a measurement chart for food consumed in Brazil. The foods will be converted into energy and nutrients using the Virtual Nutri Plus^®^ 2.0 (São Paulo, Brazil). Nutritional information from industrial food labels will also be included in the software database, as necessary, along with their nutrient composition obtained from the Brazilian food composition tables and the United States Department of Agriculture database, as appropriate [22–25]. For each patient, the average consumption of the 3 days will be considered in all the evaluated components.

### Anthropometric and body composition assessments

Anthropometric measurements such as weight, height, waist circumference (WC), and handgrip strength (HGS) will be collected. The current weight will be obtained using a digital platform scale from *Balmak*^®^ (São Paulo, Brazil), calibrated with accuracy class III, with a minimum capacity of 1 kg, and a maximum capacity of 150 kg. Height will be measured using an anodized aluminum stadiometer with a rectangular cursor from *Sanny*^®^ (São Paulo, Brazil), with wall-fixation with screws, a scale from 50 to 211 cm, and a resolution of 0.1 cm. To measure the WC, an inelastic measuring tape from *Sanny*^®^ (São Paulo, Brazil), with an extension of 200 cm and an accuracy of 1 mm will be used. The HGS, used to verify a reduction in muscle function, will be assessed based on the measurement of isomeric contraction of the hand muscles through an analog manual dynamometer from *Crown*^®^, with a scale from 0 to 100 kg and a resolution of 1 kg. The measurement will be conducted in triplicates, with the highest value being adopted between repetitions.

Body composition will be assessed at T0, T6, and T12 months after KTx using the *double-beam X-ray absorptiometry (DXA)* method, performed by the *LUNAR-GE*^®^ device *(Lunar Radiation Corporation*, *USA)*. For this, the measurements of lean mass (LM% and LM Kg), fat mass (FM% and FM/kg), body fat (%BF), bone mineral content (BMC), and fat-free mass (Kg) will be conducted.

### Assessment of the presence of sarcopenia

The presence of sarcopenia will be assessed through the recommendations of the European Group Working Sarcopenia Older People (*EGWSOP)* [26], considering the HGS data, to assess muscle mass function and body composition using DXA, verified by the Appendicular Muscle Mass Index (AMMI), to assess muscle mass.

### Quality of life assessment and physical activity

Participants in the case and control groups will be assessed for quality of life, using the generic questionnaire *Medical Outcomes Study 36-Item Short-Form Health Survey (SF-36)* translated and validated into Portuguese and applied in three periods T0, T6, and T12 after KTx [27]. The instrument consists of 36 items or questions, encompassed in eight domains and summarized into physical and mental components. The score for each domain ranges from 0 (worst health) to 100 (best health). The data analysis for tabulation will be performed through registration on an online platform through the website *qualipes*.*com*.*br* [38] by the same evaluator in all the stages of the study.

Considering that depression, anxiety, and other mental disorders can directly affect the quality of life of individuals, an interview will be conducted with the *Self-Reporting Questionnaire (SRQ-20)* [28], which is the Brazilian version of a psychiatric screening questionnaire that assesses non-psychotic mental disorders in the primary health care system, consisting of 20 items that can be answered as “Yes” or “No” with scores ranging between 7 and 8 points for men and women, respectively.

In addition to the previous parameters, the levels of physical activity of the patients will be assessed using the full version of the *International Physical Activity Questionnaire (IPAQ)*, translated and validated in Portuguese [29]. The IPAQ considers the activities carried out in the last seven days under four domains: means of transport, housework and recreation, sports, and leisure. The final score will be converted to MET-minutes/week. Assessment of physical activity will always be carried out by the same evaluator.

### Statistical analysis

The study will be analyzed by the modified intention-to-treat method, whereby all randomized patients who had at least one post-baseline measurement of proteinuria will be included in the primary analysis population. Data will be presented descriptively by means and standard deviations, or with absolute and relative frequencies, according to the respective scale of measurement. The difference between the groups in the change from baseline in the primary efficacy variable will be tested by analysis of covariance with a multiple linear regression model, with the baseline value entered as covariate, *Diabetes mellitus* entered as stratification variable, and study group as independent variable. For secondary efficacy variables, in those measured in an interval scale, the change from baseline will be compared between groups with the same method. For those variables that are related to patient sex (e.g., WC, sarcopenia), sex will be included as independent variable. For variables measured in ordinal scales, only the values observed in the last valid observation will be considered and will be compared between groups with the Wilcoxon-Mann-Whitney U test. No multiplicity correction of p-values will be applied to the secondary variables.

Statistical analyses will be performed using Stata 15.1 (Stata Corp., Collegue Station, TX). Tests with two-sided p values < 0.05 will be considered statistically significant.

### Trial status

The study started recruiting patients on May 1, 2019 and is expected to end on December 31, 2022.

## Discussion

The main clinical relevance of this randomized trial is to provide information on the impact of an individualized nutrition intervention with a defined amount of protein intake on the 25% reduction in proteinuria levels in the late post-KTx period. In addition, this study will allow to evaluate other relevant parameters related to metabolic and quality of life outcomes, since this study raises the hypothesis that this balanced diet may improve parameters of body composition, lipid and glucose profiles, as well as quality of life of patients in the post-KTx.

Patients with KTx in the present clinical trial will undergo an individualized nutritional intervention for 12 months after surgery. This period of nutritional intervention was selected based on previous studies, which demonstrated that protein intake is associated with maintenance of kidney function in the first year post-KTx [30–32]. However, studies on the assessment of the impact of dietary protein intake in post-KTx patients remain scarce and controversial [33–36].

A study conducted by Deetman et al. [33], including patients with more than 6-months post-KTx, identified that a higher protein intake would have a protective effect against renal graft failure. Another study in patients with 1 year of KTx, also suggested that the restriction of protein intake should not be advised after KTx, as low protein intake would be associated with an increased risk of mortality and graft failure [34]. Other studies that evaluated the impact of dietary intervention on the post-KTx also showed conflicting results, mainly due to limitations in their methodological designs [35, 36].

Thus, due to the lack of data with high quality of evidence on the influence of dietary intervention on post-KTx, there are no guidelines or recommendations for a specific nutritional intervention in the late post-KTx period [37]. In addition, in post-KTx, there is insufficient evidence to recommend a particular protein type (plant vs animal) in terms of the effects on nutritional status or the blood lipid profile [8].

The primary efficacy variable evaluated will be proteinuria as it is a recognized marker of kidney injury [4, 38]. Post-KTx proteinuria is associated with worse outcomes, being a strong risk factor in predicting graft survival and mortality [4]. Fernández-Fresnedo [39] also demonstrated that proteinuria at 1 year after KTx is an excellent marker of poor long-term graft prognosis.

Furthermore, research indicates that post-KTx proteinuria can be controlled through the diet [40]. A nutritional intervention to control protein intake could contribute to reduce the overload of protein levels in the glomerular capillaries and renal tubules [41–45]. As a result, hyperfiltration and glomerular pressure decrease, reducing the urinary protein excretion rate [44, 45].

Therefore, as the reduction in proteinuria may favor better kidney function, as well as a longer graft survival and reduced mortality, the present study will use proteinuria as a primary efficacy variable in order to assess whether the intervention individualized nutrition may have an antiproteinuric effect post-KTx. And, thus, assess whether the patient can benefit in guaranteeing the success of KTx and a better quality of life.

The present clinical trial was concerned with outlining exclusion criteria that could be possible biases in the assessment of the benefit of individualized nutritional intervention in the management of proteinuria in the late post-KTx period. Among the exclusion criteria are focal segmental glomerulosclerosis, systemic lupus erythematosus and IgA nephropathy, which are underlying diseases recognized for developing recurrent proteinuria after KTx due to deposition of immune complexes in the kidneys that lead to progressive renal failure [46–48]; multiple organ transplantation and a history of recurrent transplantation due to chronic rejection that may increase the risk of kidney injury and lower graft survival [49, 50]; immunosuppressive therapy with mammalian target of rapamycin (mTOR) inhibitors (everolimus or sirolimus) which has proteinuria as an adverse effect [51, 52]; and hospitalization after KTx longer than 60 days, as it is associated with complications that can lead to lower survival and graft loss [53, 54].

One of the possible limitations of this protocol will be the impossibility of blinding, which may imply risks of bias. To reduce these risks, in this study, standardized measurements and tests of anthropometric assessments, quality of life and laboratory tests will be adopted. In addition, to reduce the potential for confounding due to the variability of the measures evaluated, the same nutritionist will carry out the nutritional intervention protocol.

Given the above, the results of this study may serve as a basis for food education and nutrition actions in the late post-KTx period. Since the consumption of a controlled diet in the post-KTx may represent a benefit in the maintenance of kidney function in the long term, reducing tendencies towards negative reflexes, such as proteinuria, overweight and imbalance in lipid and glucose profile, thus collaborate with the improvement of renal graft survival and quality of life of KTx patients.

## Supporting information

S1 Checklist SPIRIT 2013 checklist. (DOC)

S1 Appendix Supplementary material. (DOCX)

S1 File (PDF)

S2 File (PDF)

## References

[1] Chan M, Patwardhan A, Ryan C, Trevillian P, Chadban S, Westgarth F, et al. Evidence-based guidelines for the nutritional management of adult kidney transplant recipients. J Ren Nutr. 2011;21: 47–51. doi: 10.1053/j.jrn.2010.10.021 21195919

[2] van den Berg E, Engberink MF, Brink EJ, van Baak MA, Gans ROB, Navis G, et al. Dietary protein, blood pressure and renal function in renal transplant recipients. Br J Nutr. 2013;109: 1463–70. doi: 10.1017/S0007114512003455 22906209

[3] Molcho M, Rozen-Zvi B, Shteinmats T, Ben Dor N, Vahav I, Nesher E, et al. Temporal changes of proteinuria after kidney transplantation: association with cardiovascular morbidity and mortality. J Nephrol. 2020;33: 1059–1066. doi: 10.1007/s40620-020-00703-6 31953621

[4] Oblak M, Mlinšek G, Kojc N, Frelih M, Buturović-Ponikvar J, Arnol M. Spot Urine Protein Excretion in the First Year Following Kidney Transplantation Associates With Allograft Rejection Phenotype at 1-Year Surveillance Biopsies: An Observational National-Cohort Study. Front Med. 2021;8: 781195. doi: 10.3389/fmed.2021.781195 34869503 PMC8635090

[5] Fry K, Patwardhan A, Ryan C, Trevillian P, Chadban S, Westgarth F, et al. Development of evidence-based guidelines for the nutritional management of adult kidney transplant recipients. J Ren Nutr. 2009;19: 101–4. doi: 10.1053/j.jrn.2008.10.010 19121782

[6] Nolte Fong J V, Moore LW. Nutrition Trends in Kidney Transplant Recipients: the Importance of Dietary Monitoring and Need for Evidence-Based Recommendations. Front Med. 2018;5: 302. doi: 10.3389/fmed.2018.00302 30430111 PMC6220714

[7] Campbell S, Pilmore H, Gracey D, Mulley W, Russell C, McTaggart S. KHA-CARI guideline: recipient assessment for transplantation. Nephrology (Carlton). 2013;18: 455–462. doi: 10.1111/nep.12068 23581832

[8] Ikizler TA, Burrowes JD, Byham-Gray LD, Campbell KL, Carrero J-J, Chan W, et al. KDOQI Clinical Practice Guideline for Nutrition in CKD: 2020 Update. Am J Kidney Dis. 2020;76: S1–S107. doi: 10.1053/j.ajkd.2020.05.006 32829751

[9] Haller MC, van der Veer SN, Nagler E V., Tomson C, Lewington A, Hemmelgarn BR, et al. A survey on the methodological processes and policies of renal guideline groups as a first step to harmonize renal guidelines. Nephrol Dial Transplant. 2015;30: 1066–74. doi: 10.1093/ndt/gfu288 25204317

[10] Martins C, Pecoits-Filho R, Riella MC. Nutrition for the post-renal transplant recipients. Transplant Proc. 2004;36: 1650–4. doi: 10.1016/j.transproceed.2004.06.065 15350441

[11] Riella MC, Martins C. Chapter 13—Nutrition and kidney transplantation [Nutrition and the kidney]. 2nd Ed. Editora Guanabara. GEN GRUPO EDITORIAL NACIONAL PARTICIPACOES S/A; 2013.

[12] Teplan V, Valkovsky I, Teplan V, Stollova M, Vyhnanek F, Andel M. Nutritional consequences of renal transplantation. J Ren Nutr. 2009;19: 95–100. doi: 10.1053/j.jrn.2008.10.017 19121781

[13] Taşdemir D, Aksoy N. Weight Gain, Energy Intake, Energy Expenditure, and Immunosuppressive Therapy in Kidney Transplant Recipients. Prog Transplant. 2020;30: 322–328. doi: 10.1177/1526924820958150 32930038

[14] Ducloux D, Kazory A, Simula-Faivre D, Chalopin J-M. One-year post-transplant weight gain is a risk factor for graft loss. Am J Transplant. 2005;5: 2922–8. doi: 10.1111/j.1600-6143.2005.01104.x 16303006

[15] Dienemann T, Ziolkowski SL, Bender S, Goral S, Long J, Baker JF, et al. Changes in Body Composition, Muscle Strength, and Fat Distribution Following Kidney Transplantation. Am J Kidney Dis. 2021;78: 816–825. doi: 10.1053/j.ajkd.2020.11.032 34352286 PMC8608755

[16] Chan A, Tetzlaff JM, Altman DG, Laupacis A, Gøtzsche PC, Krleža-Jerić K, et al. SPIRIT 2013 statement: defining standard protocol items for clinical trials. Ann Intern Med. 2013;158: 200–7. doi: 10.7326/0003-4819-158-3-201302050-00583 23295957 PMC5114123

[17] Souza KSC de. [Evaluation of nephrin and podokine as early biomarkers of proteinuria associated with the use of mTOR inhibitors in renal transplant patients]. Federal University of Rio Grande do Norte. 2019. https://repositorio.ufrn.br/bitstream/123456789/26924/1/Avalia

[18] Dallal GE. Randomization.com. [cited 5 Apr 2019]. http://randomization.com

[19] Kidney Disease: Improving Global Outcomes (KDIGO) CKD Work Group. KDIGO 2012 Clinical practice guideline for the evaluation and management of chronic kidney disease. Kidney Int. 2013;3: 1–150. 10.1038/ki.2013.243 23989362

[20] Pires AAP, Pires R Junior, de Oliveira RF. [Consistency between print and electronic IPAQ-L formats]. Brazilian J Sport Med. 2014;20: 474–479. doi: 10.1590/1517-86922014200602134

[21] Lopes RPS, Botelho RBA. [Photo Album of Food Portions]. 1th ed. São Paulo: Metha; 2008.

[22] [Nucleus of Studies and Research in Food—NEPA]. [Brazilian Food Composition Table—TACO]. 4th ed. Brazil. Campinas: NEPA—Campinas State University; 2011.

[23] TBCA. [Brazilian Food Composition Table—TBCA]. In: University of Sao Paulo (USP) [Internet]. São Paulo: Food Research Center (FoRC). Version 7.1.; 2020 [cited 26 Mar 2022]. http://www.fcf.usp.br/tbca

[24] Philippi ST. [Food Composition Table: Support for Nutritional Decision]. 6th ed. São Paulo: Manole; 2018.

[25] Department of Health Informatics. [Table of Chemical Composition of Food Version 3.0]. In: Paulista School of Medicine/Unifesp [Internet]. [cited 26 Mar 2022]. https://tabnut.dis.epm.br/

[26] Cruz-Jentoft AJ, Bahat G, Bauer J, Boirie Y, Bruyère O, Cederholm T, et al. Sarcopenia: revised European consensus on definition and diagnosis. Age Ageing. 2019;48: 601. doi: 10.1093/ageing/afz046 31081853 PMC6593317

[27] Ciconelli RM, Ferraz MB, Santos W, Meinão I, Quaresma MR. [Brazilian-Portuguese version of the SF-36. A reliable and valid quality of life outcome measure]. Brazilian J Rheumatol. 1999;39: 143–150.

[28] Mari JJ, Williams P. A validity study of a psychiatric screening questionnaire (SRQ-20) in primary care in the city of Sao Paulo. Br J Psychiatry. 1986;148: 23–6. doi: 10.1192/bjp.148.1.23 3955316

[29] Pardini R, Matsudo S, Araújo T, Matsudo V, Andrade E, Braggion G, et al. [Validation of the international physical activity level questionnaire (IPAQ -version 6): pilot study in young Brazilian adults]. Brazilian J Sci Mov. 2001;9: 45–51.

[30] Pedrollo EF, Nicoletto BB, Carpes LS, de Freitas J de MC, Buboltz JR, Forte CC, et al. Effect of an intensive nutrition intervention of a high protein and low glycemic-index diet on weight of kidney transplant recipients: study protocol for a randomized clinical trial. Trials. 2017;18: 413. doi: 10.1186/s13063-017-2158-2 28874181 PMC5585938

[31] Klaassen G, Zelle DM, Navis GJ, Dijkema D, Bemelman FJ, Bakker SJL, et al. Lifestyle intervention to improve quality of life and prevent weight gain after renal transplantation: Design of the Active Care after Transplantation (ACT) randomized controlled trial. BMC Nephrol. 2017;18: 296. doi: 10.1186/s12882-017-0709-0 28915863 PMC5599936

[32] Henggeler CK, Plank LD, Ryan KJ, Gilchrist EL, Casas JM, Lloyd LE, et al. A Randomized Controlled Trial of an Intensive Nutrition Intervention Versus Standard Nutrition Care to Avoid Excess Weight Gain After Kidney Transplantation: The INTENT Trial. J Ren Nutr. 2018;28: 340–351. doi: 10.1053/j.jrn.2018.03.001 29729825

[33] Deetman PE, Said MY, Kromhout D, Dullaart RPF, Kootstra-Ros JE, Sanders J-SF, et al. Urinary Urea Excretion and Long-term Outcome After Renal Transplantation. Transplantation. 2015;99: 1009–15. doi: 10.1097/TP.0000000000000464 25393159

[34] Said MY, Deetman PE, de Vries APJ, Zelle DM, Gans ROB, Navis G, et al. Causal path analyses of the association of protein intake with risk of mortality and graft failure in renal transplant recipients. Clin Transplant. 2015;29: 447–57. doi: 10.1111/ctr.12536 25739949

[35] Jhee JH, Kee YK, Park S, Kim H, Park JT, Han SH, et al. High-protein diet with renal hyperfiltration is associated with rapid decline rate of renal function: a community-based prospective cohort study. Nephrol Dial Transplant. 2020;35: 98–106. doi: 10.1093/ndt/gfz115 31172186

[36] Narasaki Y, Okuda Y, Moore LW, You AS, Tantisattamo E, Inrig JK, et al. Dietary protein intake, kidney function, and survival in a nationally representative cohort. Am J Clin Nutr. 2021;114: 303–313. doi: 10.1093/ajcn/nqab011 33742197 PMC8246621

[37] Baker RJ, Mark PB, Patel RK, Stevens KK, Palmer N. Renal association clinical practice guideline in post-operative care in the kidney transplant recipient. BMC Nephrol. 2017;18: 174. doi: 10.1186/s12882-017-0553-2 28571571 PMC5455080

[38] Isaksson GL, Nielsen MB, Hinrichs GR, Krogstrup N V., Zachar R, Stubmark H, et al. Proteinuria is accompanied by intratubular complement activation and apical membrane deposition of C3dg and C5b-9 in kidney transplant recipients. Am J Physiol Renal Physiol. 2022;322: F150–F163. doi: 10.1152/ajprenal.00300.2021 34927448 PMC8791842

[39] Fernández-Fresnedo G, Plaza JJ, Sánchez-Plumed J, Sanz-Guajardo A, Palomar-Fontanet R, Arias M. Proteinuria: a new marker of long-term graft and patient survival in kidney transplantation. Nephrol Dial Transplant. 2004;19 Suppl 3: iii47–51. doi: 10.1093/ndt/gfh1015 15192136

[40] Chauveau P, Combe C, Rigalleau V, Vendrely B, Aparicio M. Restricted protein diet is associated with decrease in proteinuria: consequences on the progression of renal failure. J Ren Nutr. 2007;17: 250–7. doi: 10.1053/j.jrn.2007.02.007 17586423

[41] Molina P, Gavela E, Vizcaíno B, Huarte E, Carrero JJ. Optimizing Diet to Slow CKD Progression. Front Med. 2021;8: 654250. doi: 10.3389/fmed.2021.654250 34249961 PMC8267004

[42] Kramer H. Diet and Chronic Kidney Disease. Adv Nutr. 2019;10: S367–S379. doi: 10.1093/advances/nmz011 31728497 PMC6855949

[43] Lawrence MG, Altenburg MK, Sanford R, Willett JD, Bleasdale B, Ballou B, et al. Permeation of macromolecules into the renal glomerular basement membrane and capture by the tubules. Proc Natl Acad Sci U S A. 2017;114: 2958–2963. doi: 10.1073/pnas.1616457114 28246329 PMC5358373

[44] Adeva-Andany MM, Fernández-Fernández C, Carneiro-Freire N, Vila-Altesor M, Ameneiros-Rodríguez E. The differential effect of animal versus vegetable dietary protein on the clinical manifestations of diabetic kidney disease in humans. Clin Nutr ESPEN. 2022;48: 21–35. doi: 10.1016/j.clnesp.2022.01.030 35331493

[45] Ko G-J, Kalantar-Zadeh K. How important is dietary management in chronic kidney disease progression? A role for low protein diets. Korean J Intern Med. 2021;36: 795–806. 34153180 10.3904/kjim.2021.197 PMC8273814

[46] Naciri Bennani H, Elimby L, Terrec F, Malvezzi P, Noble J, Jouve T, et al. Kidney Transplantation for Focal Segmental Glomerulosclerosis: Can We Prevent Its Recurrence? Personal Experience and Literature Review. J Clin Med. 2021;11: 1–15. doi: 10.3390/jcm11010093 35011834 PMC8745094

[47] Çeltİk A, Şen S, Tamer AF, Yılmaz M, Sarsık B, Özkahya M, et al. Recurrent lupus nephritis after transplantation: Clinicopathological evaluation with protocol biopsies. Nephrology (Carlton). 2016;21: 601–7. doi: 10.1111/nep.12657 26482014

[48] Kavanagh CR, Zanoni F, Leal R, Jain NG, Stack MN, Vasilescu E-R, et al. Clinical Predictors and Prognosis of Recurrent IgA Nephropathy in the Kidney Allograft. Glomerular Dis. 2022;2: 42–53. doi: 10.1159/000519834 35450416 PMC9017582

[49] Salahudeen AK, Hostetter TH, Raatz SK, Rosenberg ME. Effects of dietary protein in patients with chronic renal transplant rejection. Kidney Int. 1992;41: 183–90. doi: 10.1038/ki.1992.25 1593854

[50] Suárez Fernández ML, G-Cosío F. Causes and consequences of proteinuria following kidney transplantation. Nefrologia. 2011;31: 404–14. 21738244 10.3265/Nefrologia.pre2011.May.10972

[51] Fantus D, Rogers NM, Grahammer F, Huber TB, Thomson AW. Roles of mTOR complexes in the kidney: implications for renal disease and transplantation. Nat Rev Nephrol. 2016;12: 587–609. doi: 10.1038/nrneph.2016.108 27477490 PMC6553645

[52] Leal R, Tsapepas D, Crew RJ, Dube GK, Ratner L, Batal I. Pathology of Calcineurin and Mammalian Target of Rapamycin Inhibitors in Kidney Transplantation. Kidney Int reports. 2018;3: 281–290. doi: 10.1016/j.ekir.2017.10.010 30276344 PMC6161639

[53] Salazar Meira F, Zemiacki J, Figueiredo AE, Viliano Kroth L, Saute Kochhann D, D’Avila DO, et al. Factors Associated With Delayed Graft Function and Their Influence on Outcomes of Kidney Transplantation. Transplant Proc. 2016;48: 2267–2271. doi: 10.1016/j.transproceed.2016.06.007 27742276

[54] Naghibi O, Naghibi M, Nazemian F. Factors affecting length of hospitalization in kidney transplant recipients. Exp Clin Transplant. 2007;5: 614–7. 17617054

