# Supplementary material for: Effects of an individualized nutritional intervention on kidney function, body composition, and quality of life in kidney transplant recipients: Study protocol for a randomized clinical trial
Source: PLoS One. 2022 Aug 4;17(8):e0272484. doi: 10.1371/journal.pone.0272484 (PMC9352089; doi:10.1371/journal.pone.0272484)
Supplement: S1 Appendix — (DOCX) [file pone.0272484.s002.docx]

**Appendix S1: Supplementary Material**

**Table of Contents**

1. Nutritional intervention: example of eating plan 2

2. Dietary assessment: 3-day food record 4

1. Nutritional intervention: example of eating plan

**PLANEJAMENTO ALIMENTAR**


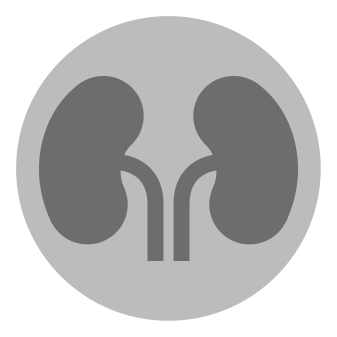


**FOOD PLANNING**

**NAME: _____________________________________________**

DATE: ___ / ___ / ____


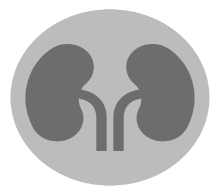


FOOD PLANNING

**NAME: ____________________________________________________________________________**

In this meal plan, you will be able to view the suggested food options per meal. The choice of food can be made by combining the food groups, as shown in the demonstration below:


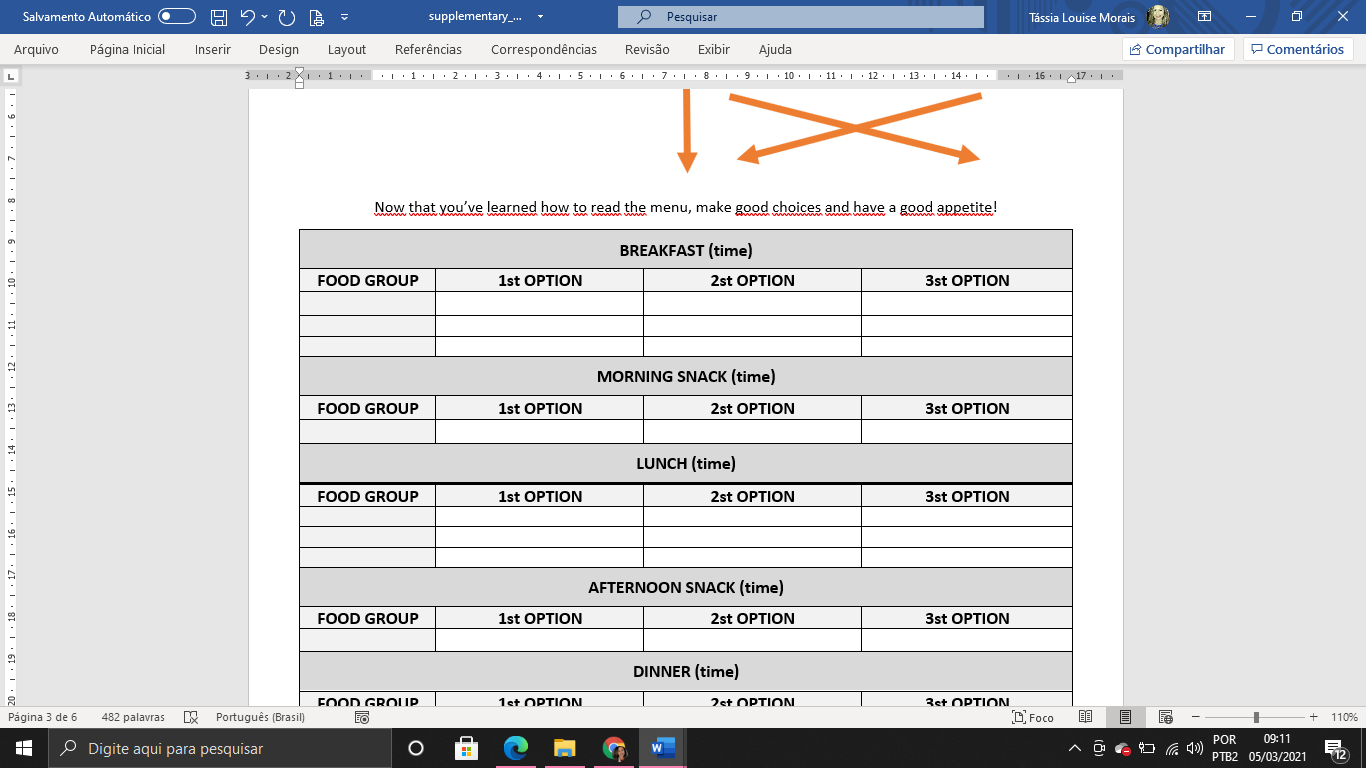


Now that you’ve learned how to read the menu, make good choices and have a good appetite!

| **BREAKFAST (time)** | | | | |
| --- | --- | --- | --- | --- |
| FOOD GROUP | 1st OPTION | 2st OPTION | | 3st OPTION |
|  |  |  | |  |
|  |  |  | |  |
|  |  |  | |  |
| **MORNING SNACK (time)** | | | | |
| FOOD GROUP | 1st OPTION | 2st OPTION | | 3st OPTION |
|  |  |  | |  |
| **LUNCH (time)** | | | | |
|  |  | |  |  |
|  |  | |  |  |
| FOOD GROUP | 1st OPTION | 2st OPTION | | 3st OPTION |
|  |  |  | |  |
|  |  |  | |  |
|  |  |  | |  |
| **AFTERNOON SNACK (time)** | | | | |
| FOOD GROUP | 1st OPTION | 2st OPTION | | 3st OPTION |
|  |  |  | |  |
|  |  | |  |  |
| **DINNER (time)** | | | | |
| FOOD GROUP | 1st OPTION | 2st OPTION | | 3st OPTION |
|  |  |  | |  |
|  |  |  | |  |
|  |  |  | |  |

2. Dietary assessment: 3-day food record

**3-DAY FOOD RECORD**

NAME: _______________________________________________DATE: ____/____/_____

**Read all instructions carefully:**

- Fill in all the information for **3 consecutive days**;

- Write down **ALL** the food and drinks consumed;

- Record the **information ate the time of the meal**, so you don’t forget it later;

- Included in your records **details of additions that have been made to foods**, such as sauces, sugars, butter, margarine, olive oil, salt and/ or other condiments;

- Don’t forget to report **the place where you are the meal**;

- **Deliver all the information filled in at the appointment** or date scheduled for reevaluation.

**How to fill in the information?**

**1) Regarding food preparation:**

Please provide details of how the food was prepared: fried, roasted, grilled, boiled or steamed, braised or poached. For example:

*Fried chicken fillet with a little soy oil*

*Mashed potatoes with cow’s milk and butter*

When it comes to a preparation, remember to list the ingredients. For example:

*Meat/ chicken lasagna witth mozzarella cheese and white sauce*

**2) Regarding the type of food:**

Whenever possible, give details of the type of food and the brand you are consuming, such as:

*Bread: brown bread, white, french or other*

*Milk: whole, skimmed, without lactose or other*

*Cheese: mozzarella, butter, ricotta, rennet or other*

*Cottage cheese: traditional or light brand “X” or other*

*Yogurt: natural, skimmed, greek “Y”-branded or other*

**3) Regarding the amount of food consumed:**

Inform in detail the quantity the of each food that was consumed, such as the type of homemade measure that was use dor the weight indicated on the food packaging. For example:

*1 serving spoon of white rice*

*1 pot of 170 grams plain yogurt*

To make it easier to fill in these quantities, consult your ‘Manual of Home Measures’.

**Space for filing:**

**DAY: ( ) 1 ( ) 2 ( ) 3 DATE: _____/_____/________**

| **Time** | **Meal location** | **Food consumed or preparation** | **Quantum** | **Type of preparation or condiments** |
| --- | --- | --- | --- | --- |
|  |  |  |  |  |
|  |  |  |  |  |
|  |  |  |  |  |
|  |  |  |  |  |
|  |  |  |  |  |
|  |  |  |  |  |
|  |  |  |  |  |
|  |  |  |  |  |
|  |  |  |  |  |
|  |  |  |  |  |
