# Supplementary material for: Effects of an individualized nutritional intervention on kidney function, body composition, and quality of life in kidney transplant recipients: Study protocol for a randomized clinical trial
Source: PLoS One. 2022 Aug 4;17(8):e0272484. doi: 10.1371/journal.pone.0272484 (PMC9352089; doi:10.1371/journal.pone.0272484)
Supplement: S1 File — (PDF) [file pone.0272484.s003.pdf]

UFRN - HOSPITAL
UNIVERSITÁRIO ONOFRE
LOPES DA UNIVERSIDADE

SUBSTANTIATED OPINION OF THE ETHICS COMMITTEE

RESEARCH PROJECT DATA

Research title: EFFECT OF NUTRITIONAL THERAPY ON THE KIDNEY FUNCTION OF INDIVIDUALS SUBMITTED TO KIDNEY TRANSPLANTATION: EVALUATION OF NUTRITIONAL STATUS AND QUALITY OF LIFE

Researcher: ADRIANA AUGUSTO DE REZENDE

Subject area:

Version: 2

CAAE: 02445018.7.0000.5292

Proposing Institution: Department of Clinical and Toxicological Analysis

Main Sponsor: Own financing

OPINION DATA

Opinion number: 3.127.266

Project presentation:

This is a controlled, randomized and randomized clinical trial with a longitudinal follow-up. An assessment will be carried out of adult individuals, aged over 18 years, who are undergoing kidney transplantation at the Onofre Lopes University Hospital (HUOL) and followed up at the HUOL Nephrology Outpatient Clinic. The sample will be obtained for convenience, with an average forecast for the collection of 40 patients, considering the data provided by the Brazilian Transplant Registry that, in the last year (2017), approximately 41 kidney transplants were performed at HUOL. The selected patients will be randomized in a simple and random way, into two groups: the case group, which will receive an individual and personalized nutritional intervention for 12 months after kidney transplantation, and the control group, which will receive standardized nutritional guidelines from the hospital of attendance. The randomized groups in the clinical trial will be evaluated at 5 times: 0 or immediate post-transplantation (period considered until the 6th week after surgery), 3, 6, 9 and 12 months after kidney transplantation. After randomization of patients, the case group will undergo a personalized nutritional intervention 60 days after kidney transplantation.

Research objective:

To evaluate the effect of a personalized nutritional intervention on kidney function, nutritional status and quality of life in individuals undergoing kidney transplantation at the University Hospital of Natal/RN.

SPECIFIC OBJECTIVES:

• Identify clinical, socioeconomic, biodemographic and dietary parameters of patients undergoing kidney transplantation;
• Assess the quality of life of patients in the case and control groups in the immediate post-transplant period and 12 months after the procedure;
• Verify food consumption in terms of energy, macronutrients, fiber and micronutrients, by applying the 3-day food record, in the case and control groups;
• Assess the nutritional status of patients in the case and control groups, using anthropometric and body composition methods using DXA, in the immediate post-transplant period and 12 months after the procedure;
• Offer nutritional guidance and personalized food planning to patients in the case group, during a period of 12 months after kidney transplantation;
• Monitor the nutritional status, kidney function and biochemical tests of patients in the case and control groups, for a period of 12 months after kidney transplantation;
• To analyze the effects of nutritional intervention and its correlations with renal function, nutritional status and quality of life of kidney transplant recipients, with emphasis on comparison between case and control groups;
• Early identification of possible complications that could impact kidney graft function and contribute to nutritional therapies.

Risk and Benefit Assessment:

After answering the pending issues, the risks and benefits are well described.

Research Comments and Considerations:

The research project is well prepared, presenting contextualized theoretical foundation, study justification and feasible methodology.

Considerations for Mandatory Subimission Terms:

The documents of mandatory presentation of the research project, Institutional declaration, Letter of consent from HUOL/UFRN, Cover sheet, informed consent form and Researcher's Identification Sheet were attached to Platform Brazil, in accordance with the requests of CEP/HUOL/UFRN and resolution 466/2012 of the National Health Council – NHC.

Recommendations:

Researchers should pay special attention to sending partial and final research reports. See models in
< http://www.ebserh.gov.br/web/huol-ufrn/cep/documentos>.

Conclusions or Pending Issues and List of Inadequacies:

After ethical analysis of the protocol in question, we concluded that it is well instructed, the requested documents were attached, the risks and benefits being well defined.

Final Considerations at the discretion of the ethics committee:

This opinion was prepared based on the documents listed below:

Document Type	Archive	Post	Author	Situation
Basic information from the projec	PB_INFORMAÇÕES_BÁSICAS_DO_PROJETO_1211114.pdf	10/01/2019 19:43:52		Accepted
Previous Opinion	resposta_parecer_cep_huol.pdf	10/01/2019 19:28:05	ADRIANA AUGUSTO DE REZENDE	Accepted
TCLE / Terms of Assent / Justification of Absence	tcle_modificado.pdf	10/01/2019 17:10:10	ADRIANA AUGUSTO DE REZENDE	Accepted
Others	cartas_de_anuencia_cep_para_envio.pdf	06/11/2018 23:17:51	ADRIANA AUGUSTO DE REZENDE	Accepted
Researchers Declaration	folha_de_identificacao_word.pdf	06/09/2018 18:25:31	ADRIANA AUGUSTO DE REZENDE	Accepted
Detailed project / Brochure Investigator	projeto_de_pesquisa_para_envio.pdf	06/09/2018 18:23:16	ADRIANA AUGUSTO DE REZENDE	Accepted
TCLE / Terms of Assent / Justification of Absence	tcle_para_envio_cep.pdf	06/09/2018 18:22:48	ADRIANA AUGUSTO DE REZENDE	Accepted
Title Page	folha_de_rosto_preenchida_e_assinada.pdf	06/09/2018 18:17:40	ADRIANA AUGUSTO DE REZENDE	Accepted

Status of Opinion:
Approved.

Needs Consideration by CONEP:
Not.

NATAL, January 31, 2019

Signed by:
jose diniz junior
(Coordinator)

Address: Avenida Nilo Peçanha, 620 - Prédio Administrativo - 1º Andar - Espaço João Machado
District: Petrópolis **CEP:** 59.012-300
State: RN **City:** NATAL
Telephone: (84)3342-5003 **Fax:** (84)3202-3941 **E-mail:** cep_huol@yahoo.com.br

SUBSTANTIATED OPINION OF THE ETHICS COMMITTEE

DATA OF EMENDATION

Research title: EFFECT OF NUTRITIONAL THERAPY ON THE KIDNEY FUNCTION OF INDIVIDUALS SUBMITTED TO KIDNEY TRANSPLANTATION: EVALUATION OF NUTRITIONAL STATUS AND QUALITY OF LIFE

Researcher: ADRIANA AUGUSTO DE REZENDE

Subject area:

Version: 3

CAAE: 02445018.7.0000.5292

Proposing Institution: Department of Clinical and Toxicological Analysis

Main Sponsor: Own financing

OPINION DATA

Opinion number: 3.440.954

Project presentation:

This is an amendment to a project already approved by this committee.

Research objective:

Study the effect of nutritional therapy on kidney transplant recipients, quality of life and nutritional status test.

Risk and Benefit Assessment:

Described.

Research Comments and Considerations:

This is an amendment with 6 modifications: Inclusion of new undergraduate and graduate students, adding a quality of life questionnaire and anthropometric measurements.

Considerations for Mandatory Subimission Terms:

All present.

Conclusions or Pending Issues and List of Inadequacies:

There are no changes in the research that did not change their ethical or moral profile in the patients studied, number of questions and measures.

This opinion was prepared based on the documents listed below:

Document Type	Archive	Post	Author	Situation
Basic information from the project	PB_INFORMAÇOES_BASICAS_1353686_E1.pdf	09/05/2019 21:43:37		Accepted
Others	formulario_emenda.pdf	09/05/2019 21:30:28	ADRIANA AUGUSTO DE REZENDE	Accepted
Detailed Project / Brochure Investigator	projeto_de_pesquisa_emenda.pdf	09/05/2019 20:47:57	ADRIANA AUGUSTO DE REZENDE	Accepted
Previous opinion	resposta_parecer_cep_huol.pdf	10/01/2019 19:28:05	ADRIANA AUGUSTO DE REZENDE	Accepted
TCLE / Terms of Assent / Justification of Absence	tcle_modificado.pdf	10/01/2019 17:10:10	ADRIANA AUGUSTO DE REZENDE	Accepted
Others	cartas_de_anuencia_cep_para_envio.pdf	06/11/2018 23:17:51	ADRIANA AUGUSTO DE REZENDE	Accepted
Declaration of Researchers	folha_de_identificacao_word.pdf	06/09/2018 18:25:31	ADRIANA AUGUSTO DE REZENDE	Accepted
Detailed Project / Brochure Investigator	projeto_de_pesquisa_para_envio.pdf	06/09/2018 18:23:16	ADRIANA AUGUSTO DE REZENDE	Accepted
TCLE / Terms of Assent / Justification of Absence	tcle_para_envio_cep.pdf	06/09/2018 18:22:48	ADRIANA AUGUSTO DE REZENDE	Accepted
Title page	folha_de_rosto_preenchida_e_assinada.pdf	06/09/2018 18:17:40	ADRIANA AUGUSTO DE REZENDE	Accepted

Status of Opinion:
Approved.

Needs Consideration by CONEP:
Not.

NATAL, July 5, 2019

Signed by:
jose diniz junior
(Coordinator)

Address: Avenida Nilo Peçanha, 620 - Prédio Administrativo - 1º Andar - Espaço João Machado
District: Petrópolis **CEP:** 59.012-300
State: RN **City:** NATAL
Telephone: (84)3342-5003 **Fax:** (84)3202-3941 **E-mail:** cep_huol@yahoo.com.br
