# Supplementary material for: Effects of an individualized nutritional intervention on kidney function, body composition, and quality of life in kidney transplant recipients: Study protocol for a randomized clinical trial
Source: PLoS One. 2022 Aug 4;17(8):e0272484. doi: 10.1371/journal.pone.0272484 (PMC9352089; doi:10.1371/journal.pone.0272484)
Supplement: S2 File — (PDF) [file pone.0272484.s004.pdf]

PARECER CONSUBSTANCIADO DO CEP

DADOS DO PROJETO DE PESQUISA

Título da Pesquisa: EFEITO DA TERAPIA NUTRICIONAL SOBRE A FUNÇÃO RENAL DE INDIVÍDUOS SUBMETIDOS AO TRANSPLANTE RENAL: AVALIAÇÃO DO ESTADO NUTRICIONAL E QUALIDADE DE VIDA

Pesquisador: ADRIANA AUGUSTO DE REZENDE

Área Temática:

Versão: 2

CAAE: 02445018.7.0000.5292

Instituição Proponente: Departamento de Análises Clínicas e Toxicológicas

Patrocinador Principal: Financiamento Próprio

DADOS DO PARECER

Número do Parecer: 3.127.266

Apresentação do Projeto:

Trata-se de um ensaio clínico controlado, randomizado e aleatorizado, de seguimento longitudinal. Será realizada avaliação de indivíduos adultos, com idade superior a 18 anos, que sejam submetidos a transplante renal no Hospital Universitário Onofre Lopes (HUOL) e acompanhados no Ambulatório de Nefrologia do HUOL. A amostra será obtida por conveniência, com previsão média para coleta de 40 pacientes, considerando os dados fornecidos pelos Registros Brasileiros de Transplante que, no último ano (2017), foram realizados cerca de 41 transplantes renais no HUOL. Os pacientes selecionados serão randomizados de forma simples e aleatória, em dois grupos: o grupo caso, que receberá uma intervenção nutricional, individual e personalizada durante 12 meses após o transplante renal, e, o grupo controle, que receberá orientações nutricionais padronizadas do hospital de atendimento. Os grupos randomizados no ensaio clínico serão avaliados em 5 momentos: 0 ou pós-transplante imediato (período considerado até a 6ª semana após a realização da cirurgia), 3, 6, 9 e 12 meses após o transplante renal. Após a randomização aleatória dos pacientes, o grupo caso será submetido a uma intervenção nutricional personalizada após 60 dias do transplante renal.

Objetivo da Pesquisa:

Avaliar o efeito de uma intervenção nutricional personalizada sobre a função renal, estado

nutricional e qualidade de vida em indivíduos submetidos ao transplante renal no Hospital Universitário de Natal/RN.

OBJETIVOS ESPECÍFICOS:
• Identificar parâmetros clínicos, socioeconômicos, biodemográficos e dietéticos dos pacientes submetidos ao transplante renal;
• Avaliar a qualidade de vida dos pacientes do grupo caso e controle no pós-transplante imediato e 12 meses após o procedimento;
• Verificar o consumo alimentar quanto aos aspectos de energia, macronutrientes, fibras e micronutrientes, mediante a aplicação do registro alimentar de 3 dias, nos grupos caso e controle;
• Avaliar o estado nutricional dos pacientes do grupo caso e controle, por meio de métodos antropométricos e de composição corporal utilizando o DXA, no pós-transplante imediato e 12 meses após o procedimento;
• Oferecer orientações nutricionais e planejamento alimentar personalizado aos pacientes do grupo caso, durante um período de 12 meses após o transplante renal;
• Acompanhar o estado nutricional, a função renal e os exames bioquímicos dos pacientes dos grupos caso e controle, durante um período de 12 meses após o transplante renal;
• Analisar os efeitos da intervenção nutricional e suas correlações com a função renal, estado nutricional e qualidade de vida dos pacientes receptores de transplante renal, com ênfase de comparação entre os grupos caso e controle;
• Identificar precocemente possíveis complicações que possam impactar a função renal do enxerto e contribuir para terapêuticas nutricionais.

Avaliação dos Riscos e Benefícios:
Após resposta à pendências, os riscos e benefícios estão bem descritos.

Comentários e Considerações sobre a Pesquisa:
O projeto de pesquisa está bem elaborado, apresentando fundamentação teórica contextualizada,justificativa do estudo e metodologia exequível.

Considerações sobre os Termos de apresentação obrigatória:
Os documentos de apresentação obrigatória projeto de pesquisa, declaração Institucional, Carta de anuência do HUOL/UFRN, Folha de rosto, TCLE e Folha de Identificação do pesquisador foram

anexados à Plataforma Brasil estando de acordo com as solicitações do CEP/HUOL/UFRN e resolução nª 466/2012 do Conselho Nacional de Saúde - CNS.

Recomendações:

Os pesquisadores devem ter especial atenção ao envio dos relatórios parcial e final da pesquisa. Ver modelos em < http://www.ebserh.gov.br/web/huol-ufrn/cep/documentos>.

Conclusões ou Pendências e Lista de Inadequações:

Após análise ética do protocolo em questão, concluímos que o mesmo se encontra bem instruído, os documentos solicitados foram anexados, estando bem definidos os riscos e benefícios.

Considerações Finais a critério do CEP:

Este parecer foi elaborado baseado nos documentos abaixo relacionados:

Tipo Documento	Arquivo	Postagem	Autor	Situação
Informações Básicas do Projeto	PB_INFORMAÇÕES_BÁSICAS_DO_PROJETO_1211114.pdf	10/01/2019 19:43:52		Aceito
Parecer Anterior	resposta_parecer_cep_huol.pdf	10/01/2019 19:28:05	ADRIANA AUGUSTO DE REZENDE	Aceito
TCLE / Termos de Assentimento / Justificativa de Ausência	tcle_modificado.pdf	10/01/2019 17:10:10	ADRIANA AUGUSTO DE REZENDE	Aceito
Outros	cartas_de_anuencia_cep_para_envio.pdf	06/11/2018 23:17:51	ADRIANA AUGUSTO DE REZENDE	Aceito
Declaração de Pesquisadores	folha_de_identificacao_word.pdf	06/09/2018 18:25:31	ADRIANA AUGUSTO DE REZENDE	Aceito
Projeto Detalhado / Brochura Investigador	projeto_de_pesquisa_para_envio.pdf	06/09/2018 18:23:16	ADRIANA AUGUSTO DE REZENDE	Aceito
TCLE / Termos de Assentimento / Justificativa de Ausência	tcle_para_envio_cep.pdf	06/09/2018 18:22:48	ADRIANA AUGUSTO DE REZENDE	Aceito
Folha de Rosto	folha_de_rosto_preenchida_e_assinada.pdf	06/09/2018 18:17:40	ADRIANA AUGUSTO DE REZENDE	Aceito

Situação do Parecer:

Aprovado

Necessita Apreciação da CONEP:
Não

NATAL, 31 de Janeiro de 2019

Assinado por:
jose diniz junior
(Coordenador(a))

Endereço: Avenida Nilo Peçanha, 620 - Prédio Administrativo - 1º Andar - Espaço João Machado
Bairro: Petrópolis **CEP:** 59.012-300
UF: RN **Município:** NATAL
Telefone: (84)3342-5003 **Fax:** (84)3202-3941 **E-mail:** cep_huol@yahoo.com.br

PARECER CONSUBSTANCIADO DO CEP

DADOS DA EMENDA

Título da Pesquisa: EFEITO DA TERAPIA NUTRICIONAL SOBRE A FUNÇÃO RENAL DE INDIVÍDUOS SUBMETIDOS AO TRANSPLANTE RENAL: AVALIAÇÃO DO ESTADO NUTRICIONAL E QUALIDADE DE VIDA

Pesquisador: ADRIANA AUGUSTO DE REZENDE

Área Temática:

Versão: 3

CAAE: 02445018.7.0000.5292

Instituição Proponente: Departamento de Análises Clínicas e Toxicológicas

Patrocinador Principal: Financiamento Próprio

DADOS DO PARECER

Número do Parecer: 3.440.954

Apresentação do Projeto:

Trata-se de uma emenda a um projeto já aprovado nesse comitê.

Objetivo da Pesquisa:

Estudar o efeito da terapia nutricional em transplantados renais. teste de qualidade de vida e estado nutricional.

Avaliação dos Riscos e Benefícios:

Descritos.

Comentários e Considerações sobre a Pesquisa:

Trata-se de uma emenda com 6 modificações: Inclusão de novos alunos de graduação e pós-graduação, acrescentar questionário de qualidade de vida e medicas antropométricas.

Considerações sobre os Termos de apresentação obrigatória:

Todos presentes.

Conclusões ou Pendências e Lista de Inadequações:

Não há pois as modificações na pesquisa não modificaram seu perfil ético ou moral nos pacientes estudados. quantidade de quesitos e medidas.

Este parecer foi elaborado baseado nos documentos abaixo relacionados:

Tipo Documento	Arquivo	Postagem	Autor	Situação
Informações Básicas do Projeto	PB_INFORMAÇÕES_BÁSICAS_1353686_E1.pdf	09/05/2019 21:43:37		Aceito
Outros	formulario_emenda.pdf	09/05/2019 21:30:28	ADRIANA AUGUSTO DE REZENDE	Aceito
Projeto Detalhado / Brochura Investigador	projeto_de_pesquisa_emenda.pdf	09/05/2019 20:47:57	ADRIANA AUGUSTO DE REZENDE	Aceito
Parecer Anterior	resposta_parecer_cep_huol.pdf	10/01/2019 19:28:05	ADRIANA AUGUSTO DE REZENDE	Aceito
TCLE / Termos de Assentimento / Justificativa de Ausência	tcle_modificado.pdf	10/01/2019 17:10:10	ADRIANA AUGUSTO DE REZENDE	Aceito
Outros	cartas_de_anuencia_cep_para_envio.pdf	06/11/2018 23:17:51	ADRIANA AUGUSTO DE REZENDE	Aceito
Declaração de Pesquisadores	folha_de_identificacao_word.pdf	06/09/2018 18:25:31	ADRIANA AUGUSTO DE REZENDE	Aceito
Projeto Detalhado / Brochura Investigador	projeto_de_pesquisa_para_envio.pdf	06/09/2018 18:23:16	ADRIANA AUGUSTO DE REZENDE	Aceito
TCLE / Termos de Assentimento / Justificativa de Ausência	tcle_para_envio_cep.pdf	06/09/2018 18:22:48	ADRIANA AUGUSTO DE REZENDE	Aceito
Folha de Rosto	folha_de_rosto_preenchida_e_assinada.pdf	06/09/2018 18:17:40	ADRIANA AUGUSTO DE REZENDE	Aceito

Situação do Parecer:
Aprovado

Necessita Apreciação da CONEP:
Não

NATAL, 05 de Julho de 2019

Assinado por:
jose diniz junior
(Coordenador(a))
